# The potential influence of melatonin on mitochondrial quality control: a review

**DOI:** 10.3389/fphar.2023.1332567

**Published:** 2024-01-11

**Authors:** Xudan Lei, Zhenni Xu, Lingxiao Huang, Yujun Huang, Siyu Tu, Lu Xu, Dengqun Liu

**Affiliations:** ^1^ Radiation Oncology Key Laboratory of Sichuan Province, Department of Experimental Research, Sichuan Clinical Research Center for Cancer, Sichuan Cancer Center, Sichuan Cancer Hospital and Institute, Affiliated Cancer Hospital of University of Electronic Science and Technology of China, Chengdu, China; ^2^ College of Basic Medicine, Chengdu University of Traditional Chinese Medicine, Chengdu, China

**Keywords:** autophagy, melatonin, mitochondrial dynamics, mitochondria-related diseases, oxidative phosphorylation

## Abstract

Mitochondria are critical for cellular energetic metabolism, intracellular signaling orchestration and programmed death regulation. Therefore, mitochondrial dysfunction is associated with various pathogeneses. The maintenance of mitochondrial homeostasis and functional recovery after injury are coordinated by mitochondrial biogenesis, dynamics and autophagy, which are collectively referred to as mitochondrial quality control. There is increasing evidence that mitochondria are important targets for melatonin to exert protective effects under pathological conditions. Melatonin, an evolutionarily conserved tryptophan metabolite, can be synthesized, transported and metabolized in mitochondria. In this review, we summarize the important role of melatonin in the damaged mitochondria elimination and mitochondrial energy supply recovery by regulating mitochondrial quality control, which may provide new strategies for clinical treatment of mitochondria-related diseases.

## 1 Introduction

Mitochondria are important organelles that not only regulate cellular energy synthesis but also determine cell fate by coordinating multiple intracellular signaling pathways (Ito and Ito, 2016; Chakrabarty and Chandel, 2021; Diehl et al., 2023). Mitochondria are dynamically changing and their quality is regulated through complex interacting processes, among which mitochondrial biogenesis, dynamics, and mitophagy are the key factors. These mitochondrial regulations are known as mitochondrial quality control (MQC). Moreover, MQC is associated with a variety of diseases, such as aging (Okatani et al., 2002; Zhang et al., 2023), cancers (de Almeida Chuffa et al., 2019; Reiter et al., 2020a; Zhang et al., 2023), cardiomyopathy (Cai et al., 2022; Zhou et al., 2023), intestinal inflammation (Motilva et al., 2011; Ma et al., 2020), ischemia/reperfusion injury (Chen et al., 2016; Bai et al., 2023), liver disease (Mauriz et al., 2007; Solís-Muñoz et al., 2011; Zhou et al., 2018a), and neurodegenerative diseases (Jauhari et al., 2020; Hossain et al., 2021; Austad et al., 2022; Xu et al., 2023; Han et al., 2023). Therefore, revealing the molecular mechanisms of MQC dysregulated may provide new prospects for therapeutic drug development in related diseases.

Interestingly, more increasing studies are showing that mitochondrial biogenesis, dynamics and autophagy are tightly regulated by melatonin, which is an evolutionarily conserved tryptophan metabolite (Tan et al., 2016a; Reiter et al., 2017; Roohbakhsh et al., 2018; Mehrzadi et al., 2021; Wu et al., 2021). Melatonin, N-acetyl-5-methoxytryptamine, is a natural endogenous hormone. In mammals, melatonin is produced in many organs such as the pineal, small intestine, retina, brain, liver, thymus, kidney, skin, and other tissues (Minich et al., 2022). In addition, melatonin is involved in the regulation of biological rhythms, free radical scavenging, anti-aging, anti-cancer, immunity and other aspects (Minich et al., 2022).

Mechanistically, melatonin modulates mitochondrial metabolism, promotes mitochondrial fusion and maintains mitochondrial oxidative stress by altering autophagy to protect against mitochondrial injury. Importantly, these protective functions of mitochondria exhibit an evolutionarily conserved pattern. Furthermore, melatonin can not only be taken up from the circulation and is produced and metabolized in mitochondria (Tan et al., 2013). An increasing body of evidence suggests that melatonin may play an important role in MQC regulation (Reiter et al., 2021; Wu et al., 2021; Reiter et al., 2022). However, the regulatory roles played by melatonin in MQC have not been fully clarified. Here, we review recent available studies and propose the possible regulatory mechanism by which melatonin maintains the homeostasis of mitochondria.

## 2 Melatonin synthesis and degradation

### 2.1 Melatonin synthesis

**FIGURE 1:**
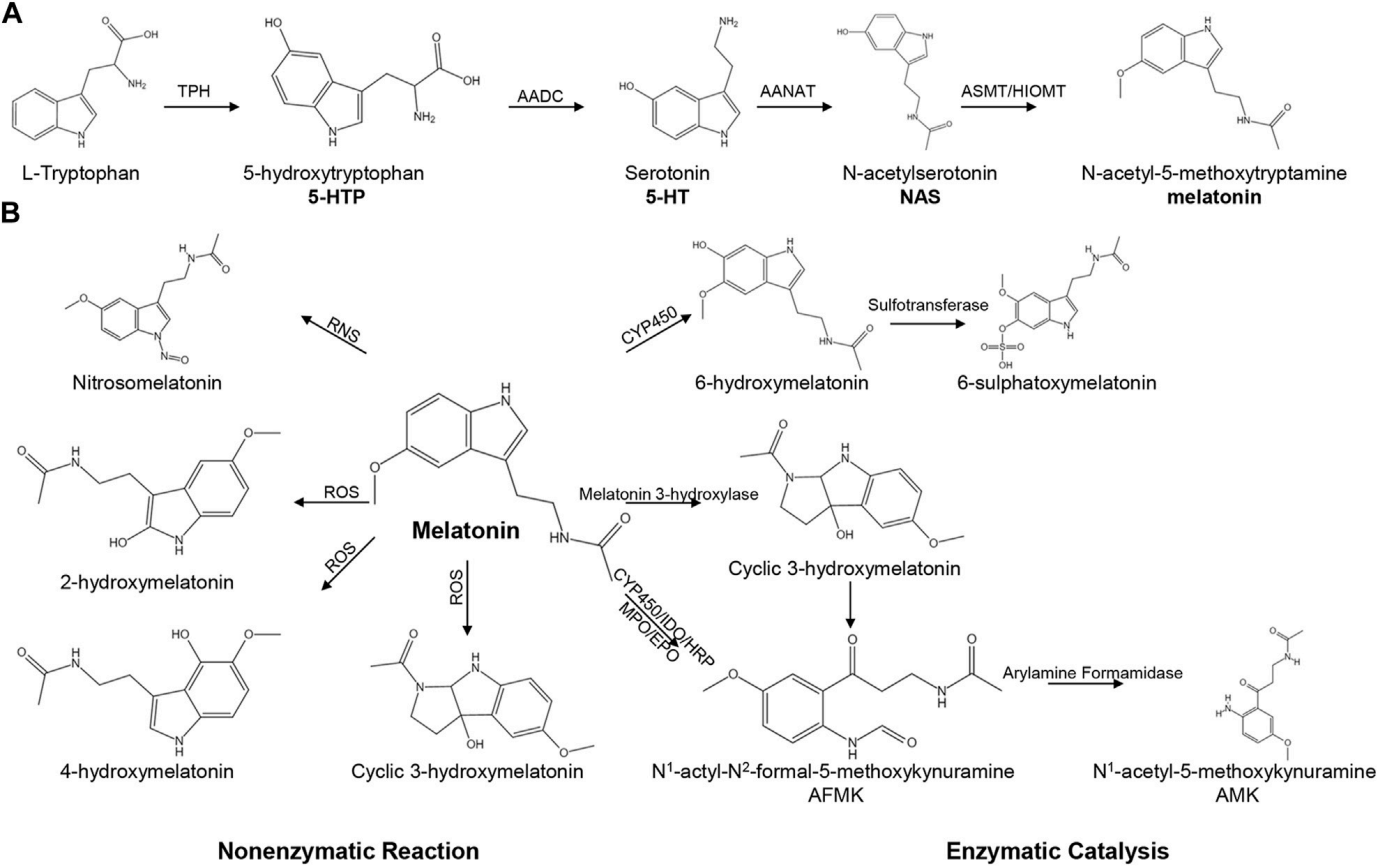
The pathways of melatonin biosynthesis and degradation. **(A)** The pathway of melatonin biosynthesis. **(B)** Melatonin degradation can be classified into non-enzymatic and enzymatic catalysis.

Tryptophan can be converted into melatonin through four enzymatic steps in pineal, retinal, intestine and other tissues (Zhao et al., 2019). Briefly, L-tryptophan is initially hydroxylated by tryptophan hydroxylase (TPH) to form 5-hydroxytryptophan (5-HTP) (Tan et al., 2016b), which is decarboxylated to produce 5-hydroxytryptophan (5-HT, serotonin) via aromatic amino acid decarboxylase (AADC) (Tan et al., 2015). Subsequently, serotonin is acetylated to produce N-acetylserotonin under the catalysis of arylalkylamine-N-acetyltransferase (AANAT). Finally, N-acetylserotonin is methylated to form 5-methoxytryptanmine (melatonin) via hydroxyindole-O-methyltransferase (HIOMT) (Figure 1A) (Tan et al., 2015; Zhao et al., 2019; Ma et al., 2020; Wu, 2021a).

TPH is the initial enzyme in the biosynthesis of melatonin and has two isoforms in mammals: TPH-1 and TPH-2 (Waløen et al., 2017). TPH-1 is predominantly expressed in endothelial cells of the gastrointestinal tract (Yadav et al., 2010), and other non-neural cell types, such as kidney (Chen et al., 2019), skin (Nowak et al., 2012; Duerschmied et al., 2013), pineal gland (Rath et al., 2016) and pituitary gland (Waløen et al., 2017). In contrast, TPH-2 is expressed in serotonergic neurons in brainstem raphe nuclei (Walther et al., 2003), the orbitofrontal cortex (Booij et al., 2012) enteric nerves (Sia et al., 2013; Waløen et al., 2017; Kulikova and Kulikov, 2019). HIOMT, as known as acetylserotonin methyltransferase (ASMT), is the catalytic enzyme in the final step in melatonin or 5-MTP biosynthesis. HIOMT has been reported to be expressed in the small intestine or colon in many species, such as humans (Chojnacki et al., 2013; Chojnacki et al., 2018), rats (Al-Ghoul et al., 2010) and sheep (Zhao et al., 2019). The expression of HIOMT was significantly increased in ulcerative colitis and lymphocytic colitis patients compared with that in healthy subjects (Chojnacki et al., 2013; Chojnacki et al., 2018). Human HIOMT is encoded by a single gene that encodes three possible isoforms: P46597-1, P46597-2 and P46597-3 (Botros et al., 2013). Among them, P46597-1 is the major isoform and the only isoform, which can synthesize melatonin. Isoform P46597-2, a truncated HIOMT, can catalyse 5-MTP synthesis (Wu, 2021b). However, P46597-3 shows no ASMT activity (Botros et al., 2013).

### 2.2 Degradation of melatonin

Melatonin can be metabolized through non-enzymatic reactions and enzymatic catalysis (Figure 1B). During the non-enzymatic process, melatonin interacts with reactive nitrogen species (RNS) to generate nitrosomelatonin under nitrosomelatonin action (Tan et al., 2007). Moreover, melatonin can react with hydroxyl radicals (^.^OH) to generate 2-hydroxymelatonin, 4-hydroxymelatonin and cyclic 3-hydroxymelatonin (Tan et al., 2007). The enzymatic catalysis of melatonin involves the cytochrome C-based pathway and the classic kynurenine pathway. The cytochrome C-based pathway is the primary metabolic pathway for melatonin, and melatonin can be catalysed to form 6-hydroxymelatonin and 6-sulphatoxymelatonin (Ma et al., 2005). In bacteria, melatonin is metabolized to form cyclic 3-hydroxymelatonin via melatonin 3-hydroxylase (Hardeland, 2017). In an inflammatory environment, melatonin is degraded through the kynurenine pathway to form N^1^-actyl-N^2^-formal-5-methoxykynuramine (AFMK) and N^1^-acetyl-5-methoxykynuramine (AMK) (Hammerle and Surawicz, 2008; Hardeland, 2017; Ma et al., 2020).

### 2.3 Uptake, synthesis and degradation of melatonin by mitochondria

Melatonin is an amphiphilic molecule which can easily interact with the phospholipid bilayers and directly cross the cell membrane (Costa et al., 1995). In addition, melatonin can also be transported into cells via binding to the membrane receptors such as melatonin receptor 1 A (MTNR1A) (Liu et al., 2016; Mayo et al., 2017), melatonin receptor 1 B (MTNR1B) (Liu et al., 2016; Mayo et al., 2017), glucose transporters (GLUT) (Hevia et al., 2015; Hevia et al., 2017; Mayo et al., 2017; Pal et al., 2023), and proton-driven oligopeptide transporter 1/2 (PEPT1/2) (Wang et al., 2011). Quinone reductase two also acts as a target of melatonin to regulate downstream signaling pathways (Nosjean et al., 2000; Boutin, 2016). In the cytoplasm, melatonin can directly bind to nuclear receptors (retinoid-related orphan nuclear hormone receptor family) (Carlberg and Wiesenberg, 1995; Karasek et al., 2003; Ma et al., 2021). Besides nucleus, melatonin also exhibits regulatory effects by modulating endoplasmic reticulum (ER) stress (Martínez-Campa et al., 2006; Wu et al., 2016; Xue et al., 2017; Fang et al., 2018; Shi et al., 2018; Lee et al., 2019; Fan et al., 2020; Mahalanobish et al., 2020; Zhang et al., 2020; Guan et al., 2023). Additionally, melatonin can be transported into mitochondria through PEPT1/2 (Huo et al., 2017). Furthermore, Xin Wang et al. found that the melatonin receptor 1 A stretched across the membrane of mitochondria in mouse brains (Wang et al., 2011). Administration of melatonin to pinealectomized rats increased the concentration of melatonin in mitochondria (Tan et al., 2013). However, the melatonin level in mitochondria was not continually increased with increases in the dose administration (Tan et al., 2013). These results suggest that mitochondria may take up melatonin to maintain their function after pinealectomy.

Mitochondria may directly synthesize and degrade melatonin. First, pinealocytes contain plenty of mitochondria, and the morphology of mitochondria changes depending on the circadian clock (Bucana et al., 1974; Calvo and Boya, 1984; Tan et al., 2016a). The mitochondrial volume in pinealocytes is significantly increased in darkness, which is the peak for the synthesis of melatonin (Calvo and Boya, 1984; Tan et al., 2016a). Second, the concentration of melatonin in mitochondria has been reported approximately 100 times higher than that in mice plasma (Martín et al., 2000a). In addition, pinealectomy would not significantly decrease the melatonin level in the cerebral cortex or liver mitochondria compared (Venegas et al., 2012). Finally, and the most importantly, AANAT, the rate-limiting enzyme in the synthetic process of melatonin, resides in the mitochondria of pinealocytes and oocytes (Kerényi et al., 1979; Sakaguchi et al., 2013; Tan et al., 2013; Coelho et al., 2015). Under the stimulation of tryptophan, mitochondria derived from oocytes produce more melatonin (Sakaguchi et al., 2013; Coelho et al., 2015). Moreover, melatonin can be converted into AFMK by cytochrome P450 in the liver mitochondria of rats (Semak et al., 2008). Taken together, these studies indicate that melatonin can be transported, synthesized, and metabolized in mitochondria, which implies that melatonin may play an important role in mitochondrial homeostasis.

## 3 Melatonin regulates mitochondrial homeostasis

### 3.1 The function of melatonin in mitochondrial biogenesis

**FIGURE 2:**
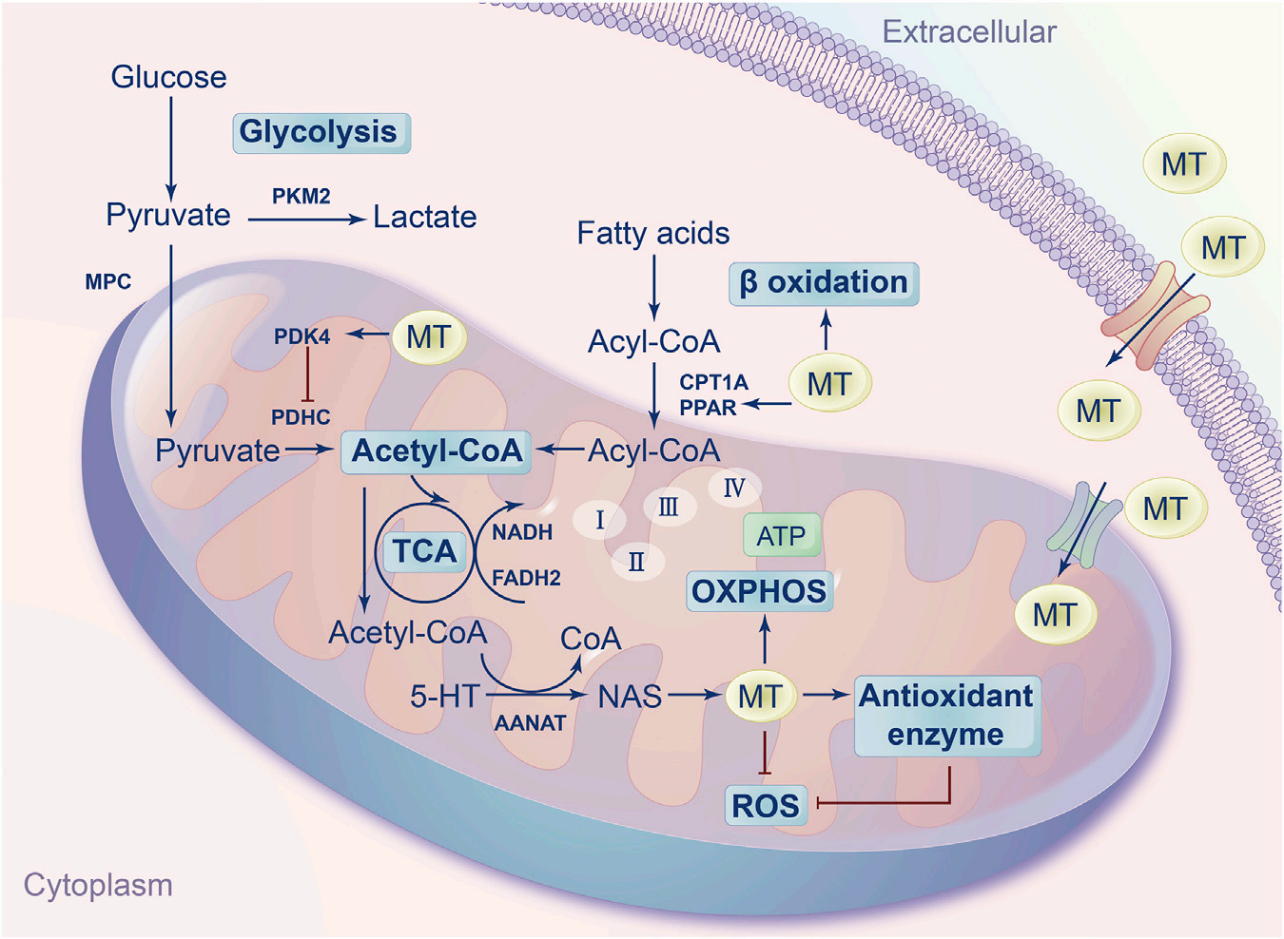
Overview of melatonin on mitochondrial metabolism and redox state. Melatonin can not only be taken from the blood into the mitochondria, but also can be synthesized in the mitochondria. Melatonin can regulate mitochondrial metabolism and redox state by regulating the concentration of acetyl-CoA, electron-transport chain, oxidative phosphorylation, reactive oxygen species, antioxidant enzymes.

Melatonin can regulate mitochondrial biogenesis by influencing metabolism and the redox state (Figure 2). First, melatonin regulates the mitochondrial concentration of acetyl-CoA to alter mitochondrial metabolism. Melatonin regulates pyruvate or fatty acid metabolism to increase the concentration of acetyl-CoA in mitochondria. For instance, melatonin increases the activity of pyruvate kinase M1/2 (PKM) to regulate glycolysis and ultimately affects the content of acetyl-CoA in mitochondria (Vakhitova et al., 2019). Moreover, melatonin activates pyruvate dehydrogenase kinase 4 (PDK4) to regulate acetyl-CoA content (Ghareghani et al., 2019). In addition, melatonin can promote fatty acid metabolism by directly enhancing *β*-oxidation or increasing the transfer of fatty acid-derived acetyl-CoA into mitochondria (Kato et al., 2015; Liu et al., 2020). In addition, melatonin synthesis can consume acetyl-CoA derived from glucose and fatty acid metabolism as required by AANAT. Taken together, these studies indicate that melatonin increases or decreases acetyl-CoA content in mitochondria to regulate mitochondrial metabolism.

Second, melatonin can enhance the activity of the electron-transport chain (ETC) and oxidative phosphorylation (OXPHOS) to regulate mitochondrial metabolism. For instance, melatonin enhanced the activities of mitochondrial complexes I and IV to protect mitochondria from ruthenium-induced injury (Martín et al., 2002). Moreover, melatonin enhanced OXPHOS and promoted adenosine triphosphate (ATP) synthesis in rat brain and liver mitochondria (Martín et al., 2000b). In addition, some studies have found that melatonin drove the switch from cytosolic glycolysis to mitochondrial OXPHOS in cancer cells (Bilska et al., 2021; Chen et al., 2021; Guerra-Librero et al., 2021). Moreover, melatonin can regulate the membrane potential of mitochondria and decrease excessive calcium levels to enhance ETC activity to increase ATP production (Xu et al., 2016).

Third, melatonin regulates the cellular redox state to influence mitochondrial metabolism. Melatonin exhibits superior antioxidant ability. Melatonin, as a major scavenger of reactive oxygen species (ROS), may play a pivotal role in protecting mitochondria from ROS-induced injury (Socaciu et al., 2020). In contrast to other antioxidants, melatonin is an amphiphilic molecule, enabling it to be distributed in aqueous or lyophobic medium (Minich et al., 2022). These specific characteristics make melatonin a broad-spectrum antioxidant. Moreover, melatonin directly scavenges various ROS, such as superoxide anions, hydroxyl radicals and hydrogen peroxide, via cascade reactions (Tan et al., 2007). In addition, melatonin increases the activity of antioxidant enzymes to eliminate ROS. Specifically, melatonin can upregulate the expression of superoxide dismutase (MnSOD), glutathione peroxidase (GSH-Px) and catalase (CAT) to prevent cell stress and injury (Fischer et al., 2013).

### 3.2 Melatonin and mitochondrial dynamics

**FIGURE 3:**
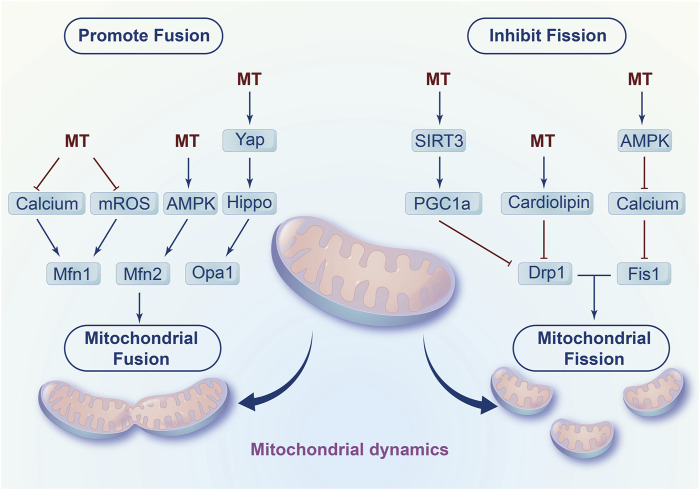
Regulation of mitochondrial fission and fusion by melatonin.

In addition to mitochondrial biogenesis, melatonin inhibits fission and promotes fusion to affect mitochondrial dynamics, which contributes to the damaged mitochondria elimination and mitochondrial energy supply recovery (Figure 3). On the one hand, melatonin increases mitochondrial fusion-related genes such as mitofusin-1 (Mfn1), mitofusin-2 (Mfn2) and optic atrophy1 (Opa1) to promote mitochondrial fusion (Singhanat et al., 2021). Mechanistically, melatonin decreases calcium accumulation and eliminates extensive ROS production to regulate mitochondrial fusion. Notably, melatonin enhanced the fusion of mitochondria by activating adenosine monophosphate activated protein kinase (AMPK) to stabilize Opa1 in myocardial ischemia/reperfusion injury (Zhang et al., 2019). In addition, studies have found that melatonin activated the Yap-Hippo pathway to increase Opa1-related fusion (Ma and Dong, 2019).

Furthermore, melatonin prevents mitochondrial fission by regulating fission-related genes such as dynamin-related protein 1 (Drp1) and Fission 1 (Fis1). For instance, in cardiac ischaemia/reperfusion induced injury, melatonin decreased the ratio of p-Drp1^ser616^/Drp1 to inhibit mitochondrial fission. Moreover, melatonin reversed methamphetamine-induced mitochondrial fission in neuroblastoma by downregulating Fis1 expression and Drp1 mitochondrial translocation (Parameyong et al., 2013; Parameyong et al., 2015). In addition, Shangcheng Xu et al. found that melatonin blocked the translocation of Drp1 from the cytoplasm to mitochondria and eliminated excess cytosolic calcium to reduce cadmium-induced neurotoxicity (Xu et al., 2016). Mechanistically, melatonin alleviated cardiac dysfunction induced by diabetes by upregulating SIRT1-PGC1α to inhibit the expression of Drp1 (Ding et al., 2018). In addition, melatonin also activates SIRT1- PGC1α to attenuate colon injury (Yao et al., 2023). Moreover, melatonin decreased excessive Ca^2+^, eliminated Ca^2+^-induced mROS and stabilized cardiolipin to prevent mitochondrial fission and swelling during oxidative injury (Peng et al., 2012). Additionally, melatonin may activate AMPK/SERCA2a to inhibit Ca^2+^ overload and thus decrease calcium-dependent xanthine oxidase and ROS levels, ultimately resulting in dephosphorylation at Ser616 inhibiting migration on the surface of mitochondria and ultimately inhibiting mitochondrial fission in LPS-induced human umbilical vein endothelial cell injury (Cui et al., 2018).

### 3.3 Melatonin regulates mitochondrial oxidative stress through autophagy

As a highly conserved mechanism, autophagy degrades misfolding proteins or damaged organelles, to fulfill the recycling of amino acids and lipids (Xu et al., 2023; Liu et al., 2023). Insufficient autophagy leads to incomplete removal of damaged organelles, which exacerbates cell or tissue injury (Lin et al., 2023). However, uncontrolled and exacerbated autophagy causes excessive degradation of cellular components, which finally induces cell death and contributes to the development of diseases (Xu X. et al., 2023; Liu et al., 2023). Therefore, the balance of appropriate autophagy is critical to maintain cellular and tissue homeostasis, and autophagy plays differential roles in different steps of disease.

**FIGURE 4:**
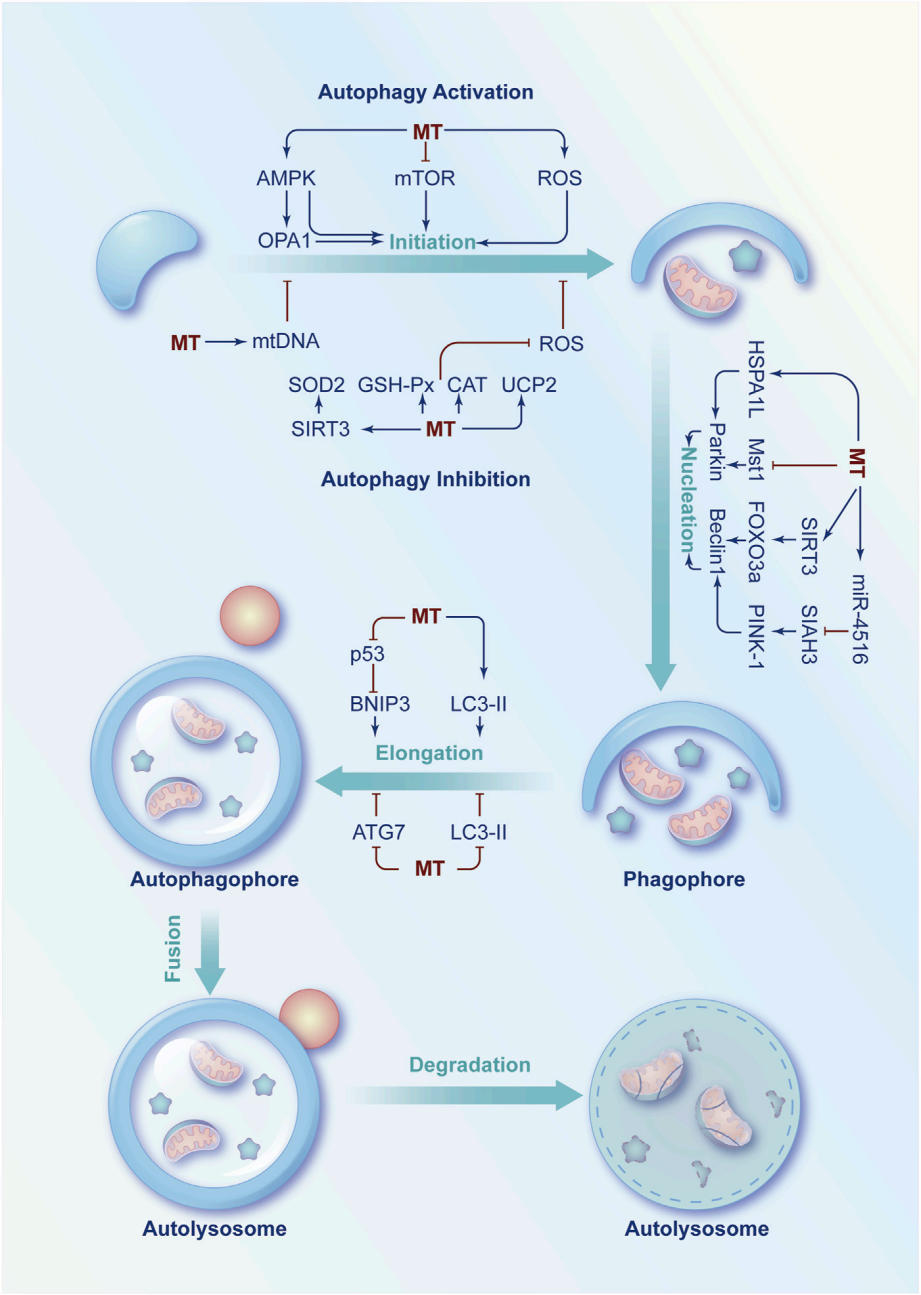
Schematic diagram of the regulatory role of melatonin in autophagy imbalance. Under physiological or pathological conditions, several signaling molecules are involved in the regulation of autophagy at the initiation, nucleation and phagophore elongation phases. Melatonin can modulate these molecules to increase or decrease autophagy rate to exert a protective effect under pathological conditions.

Autophagy plays an important role in cellular oxidative stress. As the major ROS generating organelles, mitochondria can also maintain the homeostasis of cellular oxidative stress via modulating autophagy (Scherz-Shouval and ElazarROS, 2007). ROS, as a signaling molecule, can enhance or attenuate autophagy through regulating AMPK. ROS can activate AMPK to enhance autophagy by increasing the ratio of AMP:ATP or enhancing the activity of LKB1, which is an upstream kinase of AMPK (Park et al., 2006; Fitzwalter et al., 2018). Under some circumstance, ROS can also inhibit autophagy via enhancing the activity of AKT, which is a negative regulator of AMPK (Jiang et al., 2021). Therefore, the regulation of ROS or oxidative stress on autophagy may vary depending on different circumstances (Agostini et al., 2023). Meanwhile, mitochondria are one of the main target organelles of melatonin. So that, melatonin might orchestrate autophagy to response mitochondrial oxidative stress under physiological and pathological conditions (Figure 4).

On the one hand, melatonin enhances autophagy to protect mitochondria from oxidative damage at the initiation, nucleation and phagophore elongation phases of autophagy. First, melatonin activates AMPK to regulate autophagy under different pathological conditions, such as myocardial ischemia-reperfusion injury (Zhang et al., 2019), doxorubicin-induced cardiotoxicity (Liu et al., 2018), PBDE-47 neurotoxicity (Dong et al., 2023), bone loss (McCarty et al., 2022), and lipopolysaccharide-induced blood-brain barrier injury (Wang et al., 2017). In addition, melatonin can also initiate autophagy by inhibiting the AKT/mTOR activation to enhance the therapeutic effect of rapamycin on head and neck cancer (Shen et al., 2018) or prevent hypertrophic scar (Dong et al., 2023). Moreover, melatonin combined with rapamycin and NLRP3-selective inhibitor can reduce cadmium-induced bone defects by downregulating ROS/NLRP3 signaling pathway (Hu et al., 2023). Then, melatonin modulates Beclin1 and Parkin to promote nucleation (X et al., 2023). Specifically, melatonin activates Parkin via upregulation of 70-kDa heat shock protein 1L (HSPA1L) to suppress the senescence of mesenchymal stem cells (Lee et al., 2020). Melatonin also inhibits mammalian Ste20-like kinase 1 (Mst1) to protect against diabetic cardiomyopathy (Zhang et al., 2017). Moreover, melatonin can enhance autophagy via regulating the SIRT3/FOXO3a signalling pathway to improve intervertebral disc degeneration (Chen et al., 2019). In addition, melatonin can activate the miR-4516/SIAH3/PINK-1 signalling pathway to attenuate renal fibrosis (Yoon et al., 2021). Finally, melatonin promotes the elongation of the autophagosome by relieving the repressive effect of p53 on BNIP3 (Zhou et al., 2018b) and enhancing the level of LC3-Ⅱ (Jeong et al., 2012).

On the other hand, melatonin could also inhibit autophagy to keep the mitochondrial redox state in balance in some cases. First, melatonin inhibits the initiation of autophagy by increasing mitochondrial DNA copy number or decreasing ROS levels and improve mitochondrial damage of non-alcoholic fatty liver disease or LPS-induced cardiomyopathy (Feng et al., 2013; Wang et al., 2023). In addition, melatonin might inhibit autophagy in cadmium-induced liver injury by increasing the expression and activity of silent information regulator 3 (SIRT3) to inhibit the acetylation of SOD2 and enhance the clearance ability of mitochondrial ROS (Pi et al., 2015). Moreover, melatonin can inhibit autophagy after osteoporosis by upregulating miR-224-5p to downregulate SIRT3 and AMPK (Chen and Dai, 2023). Furthermore, melatonin could elevate mitochondrial uncoupling protein 2 (UCP2) to decrease the production of mROS and inhibit autophagy in LPS-induced cardiomyopathy (Pan et al., 2018). Melatonin exerts neuroprotective effects by increasing AKT, mTOR and Unc-51 like autophagy activating kinase 1 (ULK1) to inhibit autophagy (Xiong et al., 2023). In addition, melatonin is capable to enhance the activity of SOD, CAT and GSH-Px to inhibit the formation of autophagosomes in oxidative stress-induced damaged goat spermatogonial stem cells or deoxynivalenol induced cell damage (Feng et al., 2020; Xu et al., 2023). Second, melatonin can inhibit elongation to prevent autophagy by inhibiting autophagy-related protein 7 (ATG7) activity or decreasing LC3-Ⅱ levels in kainic acid or arsenite-induced neurotoxicity (Chang et al., 2012; Teng et al., 2015).

Taken together, autophagy has a bidirectional regulatory role in cell survival or death, depending on the severity of cellular oxidative damage (Liu et al., 2023; Piletic et al., 2023). Melatonin has been proven to have a strong antioxidant effect. An intimate relationship between melatonin and autophagy has been found in various pathologies. Melatonin may modulate autophagy by regulating mitochondria and oxidative stress (Wu et al., 2021). However, the specific role of melatonin in the regulation of autophagic processes has not been extensively investigated in each pathology.

## 4 Conclusion and future prospects

In this paper, we discussed the regulatory effects of melatonin on mitochondrial quality control. Mechanistically, melatonin reprograms cellular metabolism by orchestrating the concentration of acetyl-CoA, OXPHOS levels, cellular redox state in mitochondria. Melatonin helps restore the damaged mitochondrial energy supply by regulating mitochondrial dynamics. Melatonin not only enhances mitochondrial fusion, but also has a dual role in regulating cellular autophagy. In other words, melatonin either enhances or attenuates autophagy depending on specific conditions. Under conditions of insufficient autophagy, melatonin can remove excess ROS and damaged mitochondria by upregulating autophagy. On the contrary, melatonin can down-regulate autophagy and increase cell death caused by excessive autophagy. In summary, melatonin, as a broad-spectrum antioxidant and modulator of mitochondrial activity, appears to be a promising approach for the treatment of many MQC-related injuries or diseases.

However, more basic and clinical trials are necessary to further validate the exact therapeutic effect of melatonin on MQC-related diseases (Boga et al., 2019; Reiter et al., 2020b; Melhuish Beaupre et al., 2021; Wu et al., 2021; Boc et al., 2022). For example, multiple signaling pathway are involved in aging (Martín Giménez et al., 2022; Yan et al., 2022), but the safety and efficacy of melatonin used as antioxidant therapy for clinical treatment of aging-related diseases still need further exploration (Yan et al., 2022). In addition, cutaneous cells can synthesize melatonin fight against UV-induced cutaneous damage (Slominski et al., 2008; Slominski et al., 2018; Boc et al., 2022). Is it possible to use melatonin topically to prevent the aging of skin? Why does melatonin differentially impact autophagy in different stages of aging (Hardeland, 2012), cancer (Reiter et al., 2021; Zhang et al., 2023; Li et al., 2023; Qin et al., 2023), degenerative disease (Feybesse et al., 2023; Litwiniuk et al., 2023) and ischemia/reperfusion injury (Loh and Reiter, 2021)? How does melatonin enhance or attenuate autophagy according to different stimulations or cell types? (Boga et al., 2019; Wu et al., 2021). In addition, many discoveries of melatonin are made on animals, how can they be successfully translated into MQC-related diseases in humans? The answers to these questions would not only allow us to further understand the relationship between melatonin mediated MQC and disease, but also provide more critical clues and evidence for the development of promising agents.

## Figures and Tables

**Table:** 

**AADC**	aromatic amino acid decarboxylase
**AANAT**	arylalkylamine-Nacetyltransferase
**AFMK**	N1-actyl-N2-formal-5-methoxykynuramine
**AMK**	N1-acetyl-5-methoxykynuramine
**AMPK**	adenosine monophosphate activated protein kinase
**ASMT**	acetylserotonin methyltransferase
**ATP**	adenosine triphosphate
**CAT**	Catalase
**CYP450**	cytochrome P450
**Drp1**	dynamin-related protein 1
**EPO**	eosinophil peroxidase
**ER**	endoplasmic reticulum
**ETC**	electron-transport chain
**Fis1**	Fission 1
**GSH-Px**	glutathione peroxidase
**HIOMT**	hydroxyindole O methyltransferase
**HRP**	horseradish peroxidase
**HSPA1 L**	70-kDa heat shock protein 1 L
**5-HT**	5-hydroxytryptophan
**5-HTP**	5-hydroxytryptophan
**IDO**	indoleamine 2,3-dioxygenase
**Mfn1**	mitofusin-1
**Mfn2**	mitofusin-2
**MnSOD**	superoxide dismutase
**MPO**	myeloperoxidase
**MQC**	mitochondrial quality control
**Mst1**	mammalian Ste20-like kinase 1
**MT1**	melatonin receptors 1
**mTOR**	mammalian target of rapamycin
**OH**	hydroxyl radicals
**Opa1**	optic atrophy1
**OXPHOS**	oxidative phosphorylation
**PDK4**	pyruvate dehydrogenase kinase 4
**PEPT**	oligopeptide transporters
**PKM**	pyruvate kinase M1/2
**RNS**	reactive nitrogen species
**ROS**	reactive oxygen species
**SIRT3**	silent information regulator 3
**TPH**	tryptophan hydroxylase
**UCP2**	mitochondrial uncoupling protein 2
**ULK1**	Unc-51 like autophagy activating kinase 1
